# Branched-Chain Amino Acids Combined with Exercise Improves Physical Function and Quality of Life in Older Adults: Results from a Pilot Randomized Controlled Trial

**DOI:** 10.3390/dietetics4030032

**Published:** 2025-08-01

**Authors:** Ronna Robbins, Jason C. O’Connor, Tiffany M. Cortes, Monica C. Serra

**Affiliations:** 1 Division of Geriatrics, Gerontology & Palliative Medicine, Department of Medicine, The University of Texas Health Science Center San Antonio, 7703 Floyd Curl Dr., San Antonio, TX 78229, USA;; 2 Department of Nutrition and Food Sciences, Texas Woman’s University, 304 Administration Dr., Denton, TX 76209, USA; 3 San Antonio GRECC, South Texas Veterans Health Care System, 7400 Merton Minter, San Antonio, TX 78229, USA; 4 Department of Pharmacology, The University of Texas Health Science Center San Antonio, 7703 Floyd Curl Dr., San Antonio, TX 78229, USA; 5 Division of Endocrinology, Department of Medicine, The University of Texas Health Science Center San Antonio, 7703 Floyd Curl Dr., San Antonio, TX 78229, USA; 6 Barshop Institute for Longevity & Aging Studies, Department of Medicine, The University of Texas Health Science Center San Antonio, 7703 Floyd Curl Dr., San Antonio, TX 78229, USA

**Keywords:** fatigue, branched-chain amino acids, older adults, physical functioning, exercise

## Abstract

This pilot, randomized, double-blind, placebo-controlled trial investigated the effects of branched-chain amino acids (BCAAs)—provided in a 2:1:1 ratio of leucine:isoleucine: valine—combined with exercise on fatigue, physical performance, and quality of life in older adults. Twenty participants (63% female; BMI: 35 ± 2 kg/m^2^; age: 70.5 ± 1.2 years) were randomized to 8 weeks of either exercise + BCAAs (100 mg/kg body weight/d) or exercise + placebo. The program included moderate aerobic and resistance training three times weekly. Physical function was assessed using handgrip strength, chair stands, gait speed, VO_2_ max, and a 400 m walk. Psychological health was evaluated using the CES-D, Fatigue Assessment Scale (FAS), Insomnia Severity Index (ISI), and global pain, fatigue, and quality of life using a visual analog scale (VAS). Significant group x time interactions were found for handgrip strength (*p* = 0.03), chair stands (*p* < 0.01), and 400 m walk time (*p* < 0.01). Compared to exercise + placebo, exercise + BCAAs showed greater improvements in strength, mobility, and endurance, along with reductions in fatigue (−45% vs. +92%) and depressive symptoms (−29% vs. +5%). Time effects were also observed for ISI (−30%), FAS (−21%), and VAS quality of life (16%) following exercise + BCAA supplementation. These preliminary results suggest that BCAAs combined with exercise may be an effective way to improve physical performance and reduce fatigue and depressive symptoms in older adults.

## 1. Introduction

Fatigue is a prevalent and debilitating condition in older adults, with rates ranging from 25% in primary care to as high as 98% in long-term care settings [1,2]. It can manifest as physical fatigue, characterized by reduced muscular performance and slower recovery, or as mental fatigue, marked by cognitive decline and psychological distress [3–6]. Both forms contribute to impaired function, reduced physical activity, depressive symptoms, and increased mortality risk [7–10]. These outcomes arise from multifactorial causes, including sarcopenia, cardiovascular decline, cognitive impairment, chronic inflammation, sleep disturbances, and psychological conditions such as depression and anxiety [11,12].

Exercise is one of the most effective non-pharmacological strategies to combat fatigue. Regular aerobic and resistance training have been shown to improve strength, endurance, cognitive function, and psychological well-being [13–17]. In older adults, physical activity is consistently associated with enhanced physical function, better quality of life, and reduced fatigue and depressive symptoms [17,18]. However, the increased susceptibility of older adults to exercise-induced muscle damage and delayed recovery may limit the full benefits of exercise alone [19]. This has led to interest in complementary nutritional strategies to enhance recovery and more effectively address both physical and mental fatigue.

Branched-chain amino acids (BCAAs)—leucine, isoleucine, and valine—are essential amino acids that play a key role in muscle protein synthesis [20,21]. A standardized 2:1:1 leucine:isoleucine:valine ratio is commonly used in research and commercial products, with effective doses ranging from 77 to 100 mg/kg body weight per day [22–24]. BCAAs are rapidly absorbed [25] and may be better tolerated by older adults compared to whole protein or multi-nutrient supplements, allowing for targeted delivery of anabolic amino acids such as leucine [26,27]. Several studies have explored BCAA supplementation for its potential to enhance exercise performance, reduce muscle soreness, and accelerate post-exercise recovery [22,28,29]. While some trials have reported benefits, including reduced muscle damage biomarkers and faster recovery [22,29], others have shown no significant effects [30–32]. Systematic reviews and meta-analyses have highlighted these mixed findings, attributing inconsistencies to differences in study design, participant characteristics, dosing regimens, and control for dietary protein intake [33,34]. These gaps underscore the need for well-controlled studies, particularly in older adults, to clarify the potential benefits of BCAA supplementation combined with exercise.

Despite some encouraging but inconsistent findings in younger populations, little is known about the combined effects of BCAA supplementation and exercise in older adults experiencing fatigue. This gap is especially important given the heightened vulnerability of older adults to both physical and mental fatigue, and their underrepresentation in clinical trials. Furthermore, the acute (single-dose) effects of BCAAs on fatigue remain poorly characterized in this population. Few studies have specifically targeted community-dwelling older adults with moderate fatigue or focused on mental fatigue outcomes, despite their relevance to quality of life and functional independence.

Therefore, the objective of this pilot, randomized, double-blind, placebo-controlled trial was to examine the impact of BCAA supplementation combined with exercise on physical and mental fatigue, physical performance, and self-reported quality of life in fatigued older adults. Both acute (single-dose) and longer-term (8-week) effects were assessed to provide preliminary evidence on feasibility, safety, and potential efficacy.

## 2. Materials and Methods

### 2.1. Study Design and Overview

Detailed methods for this pilot trial were published previously [35]. This study was approved by the local Institutional Review Board (UT Health San Antonio; 20220493H) and registered with ClinicalTrials.gov (Unique Identifier: NCT05484661) (https://clinicaltrials.gov/study/NCT05484661 (accessed on 3 August 2022)). The study was conducted over five phases and completed within a span of 3 to 4 months. Participants were recruited and screened in phase 1, and a total of 20 individuals were enrolled. In phase 2, baseline testing (described below) was conducted on all participants. In phase 3, the enrolled participants were randomly assigned to one of two groups: 10 participants to the exercise combined with placebo group (EX + PLA) and 10 participants to the exercise combined with BCAA supplementation group (EX + BCAAs). Further, following randomization and a 12 h fast, the acute effect of BCAA intake on fatigue was measured 30 min post-consumption of either BCAAs or maltodextrin (placebo). Phase 4 consisted of the 8-week intervention, which involved in-person exercise training three times per week and daily consumption of BCAAs or a placebo. In phase 5, all baseline (Phase 2) tests were repeated.

### 2.2. Phase 1: Recruitment and Screening

Community-dwelling older adults were recruited from San Antonio, Texas through approved registry queries, community engagement activities, and local medical and web-based advertisements including social media. Eligible participants were aged 60–80 years, with the following inclusion criteria: self-reported fatigue (≥3 on a 0–10 scale), had a body mass index (BMI) between 20 and 40 kg/m^2^, and were not engaged in structured exercise training more than twice per week. A fatigue score of ≥3 on the 0–10 numeric scale was chosen based on empirical evidence from older adult populations—where such ratings are linked to “mild” fatigue and greater functional decline, mortality risk, and reduced physical activity [36]. Additionally, a baseline medical evaluation, including medical history and physical exam, was conducted to exclude participants who were consuming protein/BCAA supplements or had medical conditions that impaired their ability to complete physical assessments. Participants with active inflammatory, autoimmune, hepatic, renal, gastrointestinal, malignant, or unstable psychiatric diseases were excluded, as were those with cognitive impairment (Montreal Cognitive Assessment (MoCA) score < 21 [37], or uncontrolled depression (Center for Epidemiological Studies Depression (CES-D) score ≥ 16 [38]. Of the 32 who signed written consent, 12 were found to be medically ineligible and 20 were enrolled.

### 2.3. Phase 2: Baseline: Assessment Measures

Eligible participants completed a series of research tests and questionnaires (described below) that assessed body composition, physical functioning, and self-report measures of psychological health. These assessments were conducted at two time points: at baseline (phase 2) and following the eight-week intervention (phase 5).

#### 2.3.1. Body Composition Measurements

BMI: Body weight (kg) was assessed using a standard physician scale, with participants wearing light clothing and no shoes. Standing height (cm) was recorded with a stadiometer during the screening process. BMI was calculated as weight (kg)/height (m^2^).

Dual-Energy X-ray Absorptiometry (DEXA): Whole-body DEXA scans were conducted using a Hologic Horizon system (Hologic Inc., Marlborough, MA, USA) to assess total body lean and body fat percentage [39]. Further, Appendicular Skeletal Muscle Index (ASMI) was calculated using the sum of the lean mass from the arms and legs (kg) and corrected for height (m^2^). Participants were classified as having sarcopenia if their ASMI was ≤7.0 kg/m^2^ for men and ≤5.5 kg/m^2^ for women [40]. For individuals whose bodies extended beyond the scanning area, a half-body scan of the right side was performed with contralateral masses estimated [41].

#### 2.3.2. Physical Fatigue and Function (Strength and Endurance)

The 3 m walk test: Usual gait speed was determined by timing how long (seconds) it took a participant to walk 3 m at their normal pace. The test was conducted twice, and the average of the two trials was recorded in meters per second (m/s). Low gait speed of <1.0 m/s was categorized as a predictor for falls [42].

Isomeric handgrip strength: Handgrip strength was evaluated in triplicate for the dominant hand using a Jamar hand digital dynamometer (Sammons Preston, Bolingbrook, IL, USA) and the average of these three measurements was calculated in kilograms. The average handgrip score was compared to age and sex normative data and a score below the normative data was categorized as low strength [43].

30 s chair stand: The chair test was used to evaluate lower extremity strength. Participants completed as many full stands as possible within 30 s with arms folded across the chest. Scored as the total number of stands in 30 s with more than halfway up at the end of 30 s scored as a full stand. Scores were compared against age-group and sex average norms [44].

Cardiopulmonary exercise test (CPET): CPET was used to evaluate maximal cardiopulmonary fitness (VO_2_max) response. VO_2_max was directly measured using a CPET conducted on an ergometer bike (Lode, The Netherlands) with breath-by-breath gas analysis using Parvo Medics (Salt Lake City, UT, USA) TrueOne 2400 metabolic measurement system. A customized protocol based on the modified Bruce protocol was used [45], with participants exercising to volitional fatigue. VO_2_max (mL/kg/min) was defined as the highest 30 s average of oxygen uptake during the final stage of the test [46]. American College of Sports Medicine’s fitness categories of superior, excellent, good, fair, poor, and very poor based upon age and sex were used to categorize VO_2_max [47].

#### 2.3.3. Self-Report Measures of Mental Fatigue and Psychological Health

Fatigue Assessment Scale (FAS): The validated 10-item scale was used to assess the severity of fatigue and its effects on daily activities. FAS scores range from 10 to 50 and a total score of <22 indicates no fatigue, and ≥22 indicates fatigue [48].

Center for Epidemiologic Studies Depression Scale (CES-D): The 20-item validated screening tool was used to assess depression symptoms. Each item on the scale offers responses ranging from 0 to 3 (0 indicating rarely or none of the time, 1 indicating some or little of the time, 2 indicating moderately or much of the time, and 3 indicating most or almost all the time). The total scores range from 0 to 60, with a standard cut-off score above 16 suggesting depression [49].

Insomnia Severity Index (ISI): The 7-item assessment tool evaluated the severity of insomnia and its impact on daily functioning. Each item was rated on a 5-point Likert scale, with total scores ranging from 0 to 28. Scores were interpreted as follows: 0–7 indicates no clinically significant insomnia, 8–14 subthreshold insomnia, 15–21 moderate insomnia, and 22–28 severe insomnia [50].

Perceived Well-Being: Global pain, fatigue, and perceived quality of life were assessed as components of perceived well-being using a 100 mm horizontal visual analog scale (VAS), a widely used and validated psychometric method for quantifying subjective experiences [51–53]. Each VAS consisted of a straight line anchored with descriptive phrases at each end (e.g., ‘no pain’ to ‘worst imaginable pain’). Participants were instructed to mark a point on the line that best reflected their current experience. Scores were determined by measuring the distance in millimeters (mm) from the left anchor to the participant’s mark, resulting in a value ranging from 0 to 100 mm. Higher scores indicated greater intensity of pain and fatigue, while for quality of life, higher scores reflected better perceived well-being [54,55].

### 2.4. Phase 3: Randomization and Acute (Single-Dose) Supplementation Intervention

Participants were randomized in a 1:1 ratio to the EX + PLA group or the EX+ BCAAs group using a computer-generated randomization sequence. To maintain allocation concealment and blinding, the study team received BCAAs and placebo supplements in identical, prepackaged containers. A study nurse not involved with data collection was the only study team member not blinded and was responsible for maintaining inventory, temperature control, labeling individual participant containers with participant ID numbers, and overseeing storage, in accordance with institutional pharmacy protocol. All participants and study personnel involved in data collection and outcome assessments were blinded to group assignments.

In phase 3, participants first completed a 400 m walk test while fasted to measure baseline endurance. The test consisted of participants walking 16 laps of a 30 m course (15 m out and 15 m back) as fast as possible on a flat surface. Additionally, participants were asked to rate how hard they felt they were working at rest using a rating of perceived exertion scale (RPE: 0–10, with 10 representing maximal exertion). Completion time ≥ 6 min (1.1 m/s) or slower during the fasted test was categorized as low physical performance [56]. To evaluate the acute (single-dose) effect of supplementation on fatigue, participants were randomized to consume either BCAAs or a maltodextrin placebo following the same blinding and allocation procedures described above.

The BCAA formulation used in this study was an over-the-counter supplement powder manufactured and distributed by Naked Nutrition© (Miami, FL, USA), containing leucine, isoleucine, and valine in a 2:1:1 ratio. Five grams of the BCAA powder contained 2500 mg of L-leucine, 1250 mg of L-isoleucine, and 1250 mg of L-valine. The placebo was produced and distributed by Nutricost© (Vineyard, UT, USA) and delivered 14 g of complex carbohydrate (maltodextrin). The powders, which appeared identical in color, were distributed in a double-blind manner, mixed with 400 mL of water and 2.5 g of a calorie-free water flavor enhancer (i.e., Crystal Light), and consumed by the participants at a dosage of 100 mg/kg/d body weight of the assigned supplement [23,24]. During this phase, supplement compliance was ensured by having participants consume the BCAAs or placebo supplement on site under direct observation by research staff. This procedure allowed for verification of adherence to the acute dosing protocol. After consuming the supplement and resting for 30 min, participants completed a second 400 m walk test. Their RPE was recorded immediately after the test.

### 2.5. Phase 4: Exercise and Supplement Intervention

#### 2.5.1. Exercise

Participants attended in-person exercise training sessions three times per week over eight weeks. The exercise training regimen was designed to deliver a moderately intensive workout that engaged the entire body. An exercise physiologist prescribed the activities, utilizing heart rate (HR) monitors to guide the process. Aerobic exercise began at a conservative level, targeting a total duration of 30 min at 40% to 50% of the maximal HR reserve, calculated using the Karvonen formula (Training HR = % (HRmax − HRrest) + HRrest. The aerobic exercise progressively increased, as tolerated, aiming for 75% to 85% of the HR reserve for a duration of 30 min. Additionally, participants engaged in resistance training using resistance bands, which targeted five major muscle groups. Participants performed chest press, knee extension, leg curl, row, and bicep curl for 15 repetitions for 2 sets and until exhaustion on the third set. Resistance gradually increased when participants could complete 20 repetitions on the third set to account for strength gains.

#### 2.5.2. BCAAs and Placebo Consumption

In addition to exercise, participants continued an 8-week daily regimen of either BCAAs or placebo at a dosage of 100 mg/kg body weight [23,24]. The supplement was dissolved daily in 400 mL of water with the addition of 2.5 g of a calorie-free water flavor enhancer. On exercise days, the supplement was consumed immediately after the exercise session under the supervision of the study staff, who also recorded its distribution and consumption. The supplement for non-exercise days was provided in prepackaged containers with water flavor enhancers and distributed weekly; participants were instructed to take it between 9 AM and 10 AM and to log their intake on a provided spreadsheet.

### 2.6. Phase 5: Post-Intervention Testing

Post-intervention (Phase 5), all tests performed in Phase 2 were repeated as outlined in Section 2.3, and all assessments were completed within 5 days of the final exercise session. Participants continued to take the supplements daily until all post-testing procedures were completed.

### 2.7. Statistical Analysis

Descriptive statistics are reported as mean ± SEM for continuous variables and as frequencies and percentages for categorical variables. Pre-intervention values were compared using independent t-tests for continuous variables or Fisher’s exact test for categorical variables. Data were assessed for normality using the Shapiro-Wilk test prior to conducting parametric tests. Repeated measures ANOVA was used to evaluate changes over time between groups. Paired t-tests were used to assess within-group changes from baseline to post-intervention. Subjective fatigue response to the 8-week BCAAs intervention was designated as the primary endpoint, while changes in body composition and other physiological and physiological measures were considered secondary endpoints. These tests were two-tailed, and *p* < 0.05 was considered statistically significant. Given the pilot nature of this study, *p*-values are presented for hypothesis-generating purposes.

## 3. Results

### 3.1. Characteristics

Out of the 20 participants who underwent pre-intervention testing, one participant withdrew from the intervention due to disliking the mouthfeel of the BCAA supplement. Therefore, the data reported include 9 participants in the EX + BCAAs group and 10 participants in the EX + PLA group who completed the intervention (Table 1). The participants were of diverse ethnic backgrounds: Non-Hispanic White (53%), Hispanic White (42%), and Non-Hispanic Black (5%). Despite randomization, there was a noticeable difference in the prevalence of females between groups, with 44% in the EX + BCAAs group compared to 80% in the EX + PLA group (*p* < 0.01). Additionally, the total body lean mass at baseline was on average 1.6 kg higher in the EX + BCAAs group compared to the EX + PLA group (*p* < 0.01). No other significant differences were observed between groups at baseline in terms of body composition. The majority of participants were classified as obese, with an average BMI of 34 ± 2 kg/m^2^, and 16% of participants had sarcopenia.

Psychological health data at baseline did not show any significant differences between groups (Table 2). None of the participants had depression at baseline. Regarding insomnia, 47% had no insomnia, 26% had subthreshold insomnia, and 26% had moderate-severity insomnia, with no cases of severe insomnia. According to the FAS criteria, 8 participants had no fatigue, while 11 had fatigue. The average fatigue measured by VAS was approximately 38 mm (with a range of 24–86 mm), pain was around 31 mm (with a range of 2–85 mm), and quality of life was about 66 mm (with a range of 15–85 mm).

Baseline physical function data did not differ between groups (Table 3). Participants generally exhibited poor physical fitness and function, with 63–95% at risk for falls based on normative/cut-off values for handgrip strength, usual gait speed, and 30 s chair stands. All participants were categorized as very poor in terms of VO_2_max.

### 3.2. Intervention Effects

#### 3.2.1. Effects of Acute BCAA Supplementation

**Figure 1.**
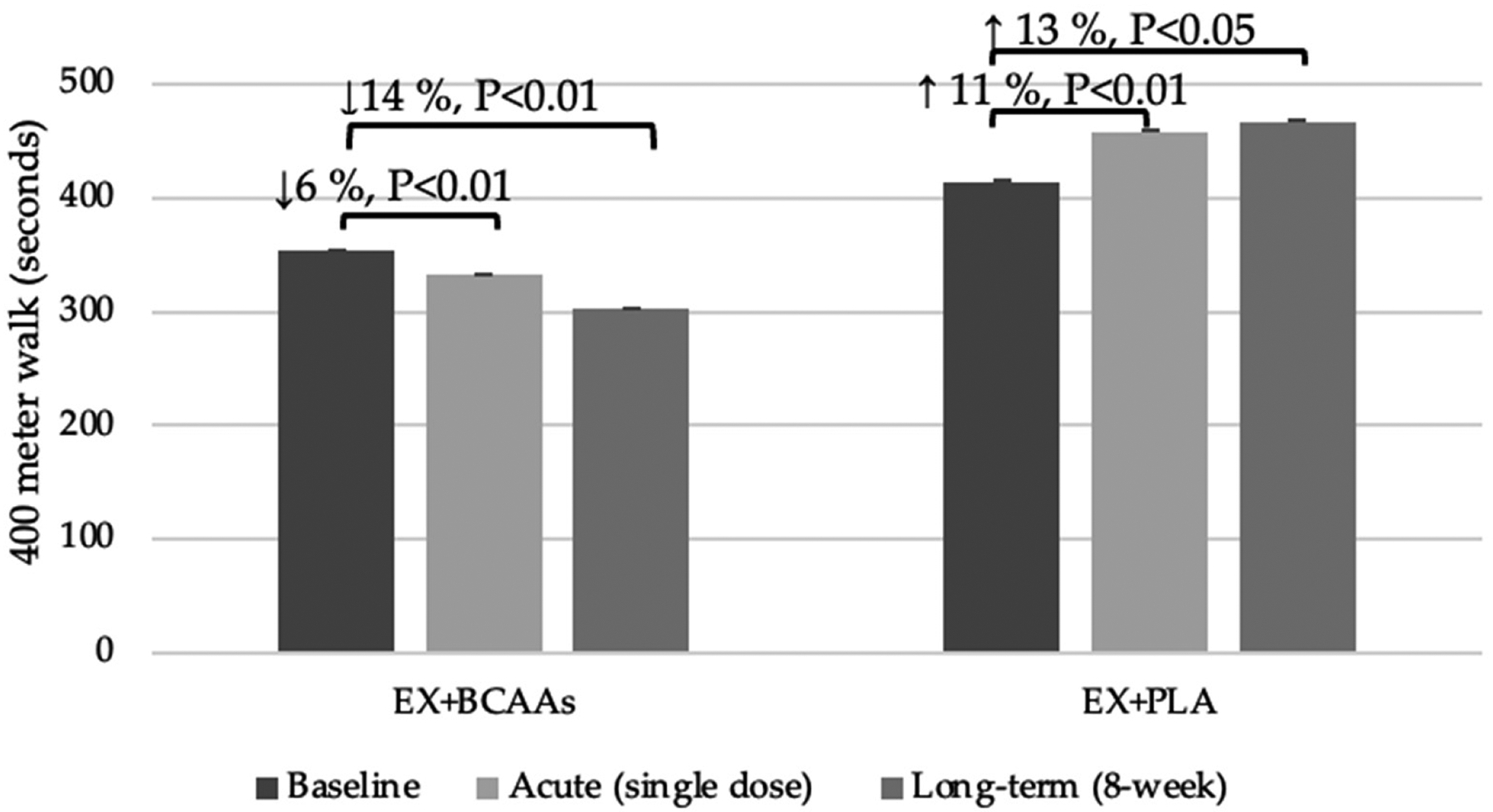
Effects of the Interventions on 400-Meter Walk Time Following Acute and Long-Term Treatment. Figure 1 Legend: 400 m walk times (mean ± SEM) are shown at baseline, after a single “acute” dose of 100 mg/kg BCAAs, and after 8 weeks of BCAA supplementation plus exercise (EX + BCAAs) vs. placebo supplementation plus exercise (EX + PLA).

Results of the acute BCAA supplementation on the 400 m walk test may be viewed in Figure 1. At baseline, the EX + PLA group took longer (an additional 61 s) to complete the walk (*p* = 0.04). Following a single dose of BCAA supplementation, the EX + BCAAs group showed a statistically significant 6% improvement in the time to complete the 400 m walk (mean change: −21.0 ± 4.3 s; *p* < 0.01 within group), whereas the EX + PLA group experienced an 11% worsening of performance (mean change: +43.6 ± 11.9 s; *p* < 0.05 within group). This resulted in a significant group*time interaction (*p* < 0.01). Regarding perceived exertion (RPE), the EX + PLA group had a slightly higher baseline RPE (EX + BCAAs: 4.3 ± 0.7 vs. EX + PLA: 5.7 ± 0.7; *p* = 0.46). After the acute supplementation and the second 400 m walk, RPE remained higher in the EX + PLA group. However, the change in RPE (pre- to post-supplementation walk) did not differ significantly between groups (EX + BCAAs: −0.86 ± 1.03 vs. EX + PLA: −0.67 ± 0.85; *p* = 0.88).

#### 3.2.2. Effects of 8-Week BCAA Supplementation and Exercise Intervention

Compliance with the exercise sessions (78% attendance to available sessions in the EX + BCAAs group vs. 79% in the EX + PLA group) and daily consumption of the supplements (96% in the EX + BCAAs group vs. 95% in the EX + PLA group) were similar between groups. Results of the longer-term (8-week) intervention on body composition may be viewed in Table 1. Body weight and BMI did not change during the intervention; however, there was an overall time effect (*p* = 0.04) such that across the intervention, ASMI increased ~4% (mean change: +0.3 ± 0.1 kg; *p* < 0.05). No changes in total body lean mass were observed; however, there was an overall time effect (*p* < 0.01) on total body fat percentage across the intervention, with groups experiencing an average decline of 0.7 ± 0.3%.

Longer-term intervention effects on psychological health may be viewed in Table 2. There was a significant group*time interaction for both CES-D score (*p* < 0.01) and fatigue score measured by VAS (*p* = 0.03). CES-D scores decreased by 29% in the EX + BCAAs group (mean change: −2.6 ± 1.5; *p* < 0.01 within group), but increased by 91% in the EX + PLA group (mean change: +5.1 ± 1.1; *p* < 0.01 within group). Fatigue scores measured via VAS decreased by 45% in the EX + BCAAs group (mean change: −12.9 ± 5.7 mm; *p* < 0.01 within group), but increased by 5% in the EX + PLA group (mean change: +1.9 ± 3.3 mm; *p* > 0.05 within group). Time effects were observed for the ISI (*p* < 0.05), FAS (*p* < 0.01), and VAS QOL (*p* = 0.04) scores, such that on average across the groups, ISI decreased by 30%, FAS decreased by 20%, while quality of life measured via VAS increased by 17%. No changes in MoCA score or pain score measured via VAS were observed.

Several group*time interactions were observed with regard to physical functioning following the longer-term intervention (Table 3). Handgrip strength (*p* = 0.03) improved by 16% in the EX + BCAAs group (mean change: +4.6 ± 1.1 kg; *p* < 0.01 within group), but declined by 6% in the EX + PLA group (mean change: −1.4 ± 2.2 kg; *p* > 0.05 within group). The usual gait speed (*p* = 0.06) tended to improve by 13% in the EX + BCAAs group (mean change: +0.14 ± 0.06 m/s; *p* < 0.05 within group), but declined by 2% in the EX + PLA group (mean change: −0.02 ± 0.05 m/s; *p* > 0.05 within group). The number of chair stands (*p* < 0.01) increased by 22% in the EX + BCAAs group (mean change: +3.4 ± 0.9 stands; *p* < 0.01 within group), while only increasing by 8% in the EX + PLA group (mean change: +1.1 ± 0.6 stands; *p* > 0.05 within group). There was a time effect (*p* = 0.01) observed for VO_2_max, such that across the interventions, VO_2_max improved by 27% (mean change: +4.0 ± 1.3 ml/kg/min; *p* < 0.01).

Effects of the longer-term BCAA supplementation on the 400 m walk test may be viewed in Figure 1. There was a significant group*time interaction (*p* < 0.01) such that the time to complete the 400 m walk test improved by 14% in the EX + BCAAs group (mean change: −51.0 ± 11.7 s; *p* < 0.01 within group), but declined by 13% in the EX + PLA group (mean change: +52.0 ± 12.3 s; *p* < 0.01 within group).

## 4. Discussion

This pilot, randomized, double-blind, placebo-controlled trial provides preliminary evidence that BCAA supplementation combined with exercise may mitigate both physical and mental fatigue to a greater extent than exercise alone in older adults. Significant improvements with BCAA supplementation and exercise were observed in several key domains including handgrip strength, chair stands, gait speed, 400 m walk time, and self-reported fatigue and depressive symptoms. While our findings indicate several promising effects of the combined intervention on physical function and self-reported fatigue, they should be interpreted with caution due to the exploratory nature of the study and the small sample size.

### 4.1. Physical Fatigue and Function

One of the most notable findings of the current study was the greater improvement in physical functioning in the EX + BCAAs group compared to the EX + PLA group. While the EX + BCAAs group exhibited marked improvements in physical fatigue and functioning, in contrast, the EX + PLA group demonstrated only minimal improvements or even slight declines. Notably, the improvement in chair stands in the EX + BCAAs group was nearly three times greater than that observed in the EX + PLA group, suggesting enhanced muscular endurance and strength in participants receiving BCAA supplementation. These functional gains are clinically meaningful, as reduced gait speed and poor 400 m walk performance are established predictors of falls, hospitalization, loss of independence, and mortality in older adults [57–59].

Our findings align with previous research that has demonstrated BCAA supplementation can enhance muscle strength, reduce muscle damage and soreness, and improve post-exercise recovery [60,61]. For example, Ko et al. [60] reported improvement in gait speed and grip strength after 5 weeks of BCAA supplementation in older adults with sarcopenia. Similarly, VanDusseldorp et al. [61] demonstrated that 8 days of BCAA supplementation in resistance-trained men modestly reduced markers of muscle damage and perceived soreness and supported recovery of isometric strength. However, other studies, including those reviewed by Martinho et al. [33] and Plotkin et al. [32], have found limited or no effects of BCAA supplementation on functional outcomes, especially when background dietary protein intake is adequate. These results highlight the need for further research to clarify the conditions under which BCAA supplementation provides functional benefits, particularly in older adults with suboptimal muscle function or dietary protein intake.

### 4.2. Mental Fatigue and Psychological Health

Beyond physical performance, we observed beneficial effects of BCAA supplementation combined with exercise on psychological health. Notably, the EX + BCAAs group showed greater improvements in self-reported fatigue (by VAS) and depression scores compared to the EX + PLA group. These findings are consistent with prior studies demonstrating psychological benefits of BCAA supplementation, particularly in clinical populations [26,27,62]. For instance, a randomized trial by Matsuda et al. [26] reported significant reductions in depressive symptoms after 24 weeks of BCAA supplementation in older adults with diabetes. Similarly, BCAA supplementation improved psychological outcomes and mental fatigue in patients with cirrhosis [63], heart failure [62], and cancer [64]. Our study extends this evidence to community-dwelling older adults, demonstrating that BCAAs plus exercise, may help reduce fatigue and alleviate depressive symptoms.

In contrast, several other psychological health outcomes—including insomnia and quality of life—demonstrated significant main effects of time but not significant group*time interactions. These improvements, including a 15–20% improvement in insomnia and quality of life scores, likely reflect the general benefit of physical activity or study engagement rather than specific effects of BCAAs. Similarly, the fact that total body fat percent decreased over time suggests the intervention as a whole (e.g., exercise, study participation) was effective. However, because there was no significant difference between groups, this benefit cannot be attributed specifically to BCAA supplementation. It is also worth noting that cognitive function (measured via MoCA) and pain (measured via VAS) did not improve significantly, likely due to ceiling and floor effects, respectively, given high baseline cognitive scores and low pain levels in our cohort.

### 4.3. Mechanistic Insights

Without direct measures of inflammation, muscle damage, or related biomarkers, definitive conclusions about the physiological mechanisms underlying the effects on physical and mental fatigue cannot be drawn. However, the following pathways are offered as biologically plausible and hypothesis-generating mechanisms for the clinical improvements observed, based on existing literature.

The benefits of BCAA supplementation on physical function may result from both long-term anabolic adaptations and short-term recovery effects. Over time, BCAAs—particularly leucine—are known to activate the mammalian target of rapamycin (mTOR) signaling pathway, which promotes protein synthesis and reduces protein breakdown [65,66]. This anabolic effect supports muscle maintenance and helps prevent muscle wasting, a critical factor in preserving physical function in older adults [67]. In the short term, BCAAs may aid post-exercise recovery by reducing muscle protein breakdown and exercise-induced muscle damage. For example, acute BCAA ingestion has been shown to activate the mTOR pathway and suppress muscle catabolism even in the absence of other amino acids [61,65]. A randomized controlled trial by Waldron et al. [68] demonstrated that a single pre-exercise dose of BCAA supplementation (0.087 g/kg body mass in a 2:1:1 leucine:isoleucine:valine ratio) enhanced isometric strength, reduced perceived muscle soreness, and improved performance in resistance-trained athletes [68]. Together, these long- and short-term effects may contribute to improved physical performance and reduced fatigue. BCAAs may also contribute to reduced mental fatigue and improved psychological well-being through central mechanisms. One proposed pathway is the ‘BCAAs–serotonin hypothesis,’ which suggests that BCAAs compete with tryptophan for transport across the blood–brain barrier, potentially lowering central serotonin synthesis and delaying mental exhaustion [69]. Additionally, BCAAs could modulate the kynurenine pathway, potentially reducing production of neurotoxic metabolites linked to fatigue and mood disturbances [70–73]. While these mechanisms were not directly assessed in this study, they highlight avenues for future work exploring how BCAAs may support both physical and psychological health in older adults.

Although these pathways were not directly assessed in this study, they provide a framework for future research aimed at elucidating the mechanisms by which BCAA supplementation may enhance both physical and mental health in older adults.

### 4.4. Limitations and Future Research

While the results of this study are promising, several limitations must be acknowledged. First, we did not adjust for multiple comparisons due to the exploratory nature of the study, the small sample size, and the pre-specified hypotheses, acknowledging the need for cautious interpretation and future validation. The small sample size and the pilot nature of the study restrict the generalizability of the results. The potential for Type I error increases with multiple comparisons, especially in a study of this scale. Further, an imbalance in the sex distribution between the two groups may be a limitation, as differences in male and female physiology could influence the metabolic response to supplementation and confound the results. Future studies also should incorporate comprehensive dietary assessments (e.g., dietary recalls) to better control for and evaluate the potential influence of background diet on intervention outcomes. As such, the results are considered preliminary and should be interpreted with caution. Future studies should incorporate appropriate adjustments for multiple comparisons within their statistical analysis plans. It is possible that a longer intervention, higher doses of BCAAs, and/or a larger, more diverse sample are needed to confirm these results and enhance their applicability to broader populations.

Another limitation is the absence of direct measurements of inflammation and kynurenine metabolites, which would have provided deeper mechanistic insights into the observed effects of BCAA supplementation on fatigue. Additional research is also needed to determine the mechanisms by which BCAAs may impact symptoms of major depression, mental fatigue, and overall psychological health.

## 5. Conclusions

This study contributes to the growing body of BCAA research by focusing on an understudied population: community-dwelling older adults with self-reported fatigue and reduced physical function. While BCAAs have been widely examined in younger, athletic, or clinical populations, few trials have explored their effects in aging adults experiencing fatigue—a common yet often overlooked concern. Our findings demonstrate preliminary improvements in both objective physical performance and subjective fatigue, helping to address this gap. The inclusion of participants with elevated BMI and suboptimal functional status further enhances the clinical relevance, as these characteristics are prevalent among older adults at risk for physical decline. Collectively, these results extend existing literature and provide a foundation for future, adequately powered studies investigating fatigue and recovery strategies in aging populations.

In conclusion, our study offers preliminary evidence that combining BCAA supplementation with regular exercise may help reduce mental and physical fatigue in older adults. While these initial findings suggest potential benefits beyond exercise alone, the small sample size and exploratory nature of this pilot trial warrant cautious interpretation and further investigation in larger, well-powered studies is needed. Additionally, more research is required to elucidate the specific mechanisms involved and to determine the long-term benefits of BCAA supplementation on fatigue in older adults.

## Figures and Tables

**Table 1.** Demographic and Body Composition Effects Across the 8-week Interventions.

	EX + BCAAs (*N* = 9)										EX + PLA (*N* = 10)													
	Pre-Intervention			Post-Intervention			Change				Pre-Intervention			Post-Intervention			Change				Baseline Group Comparison	Group × Time Effect	Overall Group Effect	Overall Time Effect
	Mean	±	SEM	Mean	±	SEM	Mean	±	SEM	*p*-Value *	Mean	±	SEM	Mean	±	SEM	Mean	±	SEM	*p*-Value *	*p*-Value	*p*-Value	*p*-Value	*p*-Value
Age (years)	70.4	±	1.2		-			-			70.7	±	1.1		-			-						
Weight (kg)	84.3	±	6.5	83.5	±	6.5	−0.78	±	0.43	0.11	94.0	±	4.9	94.5	±	5.1	0.42	±	0.47	0.39	0.75	0.10	0.23	0.58
BMI (wt/ht^2^)	32.2	±	3.4	31.9	±	3.4	−0.3	±	0.1	0.10	36.4	±	1.6	36.6	±	1.6	0.2	±	0.2	0.45	0.16	0.11	0.26	0.74
Total Body Fat (%)	33.7	±	3.3	32.8	±	3.5	−0.90	±	0.64	0.20	47.1	±	1.2	46.5	±	1.2	−0.054	±	0.30	0.11	0.22	0.62	0.06	<0.01
Total Body Lean Mass (kg)	48.6	±	2.6	49.0	±	2.9	0.42	±	0.64	0.53	47.0	±	2.9	47.8	±	3.1	0.79	±	0.40	0.08	<0.01	0.13	0.73	0.63
ASMI (kg/m^2^)	7.90	±	0.45	8.11	±	0.69	0.24	±	0.16	0.43	7.45	±	0.48	7.85	±	0.57	0.40	±	0.16	0.04	0.95	0.28	0.63	0.04

EX: exercise; BCAAs: branched-chain amino acids; PLA: placebo; SEM: standard error of the mean; BMI: Body Mass Index; ASMI: appendicular skeletal muscle index.

* Within-group pre- to post-intervention comparison.

**Table 2.** Effects of the 8-week Interventions on Psychological Well-being.

	EX + BCAAs (*N* = 9)										EX + PLA (*N* = 10)													
	Pre-Intervention			Post-Intervention			Change				Pre-Intervention			Post-Intervention			Change				Baseline Group Comparison	Group × Time Effect	Overall Group Effect	Overall Time Effect
	Mean	±	SEM	Mean	±	SEM	Mean	±	SEM	*p*-Value *	Mean	±	SEM	Mean	±	SEM	Mean	±	SEM	*p*-Value *	*p*-Value	*p*-Value	*p*-Value	*p*-Value
CES-D score	9.0	±	1.3	6.4	±	1.3	−2.6	±	1.5	0.13	5.6	±	1.7	10.7	±	1.6	5.1	±	1.4	0.01	0.13	<0.01	0.82	0.25
MoCA score	26.4	±	0.8	27.3	±	1.1	0.9	±	1.0	0.43	27.9	±	0.6	27.4	±	0.9	−0.5	±	0.8	0.53	0.14	0.29	0.42	0.77
ISI score	10.6	±	2.0	9.0	±	1.8	−1.6	±	1.4	0.31	9.4	±	2.4	5.3	±	1.6	−4.1	±	2.1	0.09	0.58	0.36	0.36	<0.05
FAS score	19.5	±	1.7	17.3	±	1.1	−2.3	±	0.9	0.04	25.8	±	2.9	18.9	±	1.9	−6.9	±	2.4	0.02	0.28	0.11	0.15	<0.01
Fatigue (mm)ˆ	28.6	±	4.7	15.7	±	2.9	−12.9	±	5.7	0.06	35.3	±	6.9	37.2	±	7.8	1.9	±	3.3	0.58	0.96	0.03	0.12	0.10
Pain (mm)ˆ	21.9	±	6.8	13.1	±	4.7	−8.7	±	4.6	0.11	29.7	±	7.6	30.7	±	8.1	1.0	±	3.3	0.77	0.53	0.10	0.22	0.18
QOL (mm)ˆ	68.3	±	8.9	85.9	±	1.4	17.6	±	9.3	0.11	69.7	±	8.3	75.3	±	5.0	5.7	±	5.9	0.36	0.58	0.28	0.58	0.04

EX: exercise; BCAAs: branched-chain amino acids; PLA: placebo; SEM: standard error of the mean; CES-D: Center for Epidemiological Studies Depression; MoCA: Montreal Cognitive Assessment; ISI: Insomnia Severity Index; FAS: Fatigue Assessment Scale; QOL: quality of life.

ˆ Measured via visual analog scale.

* Within-group pre- to post-post-intervention comparison.

**Table 3.** Effects of the 8-week Interventions on Physical Functioning.

	EX + BCAAs (*N* = 9)										EX + PLA (*N* = 10)													
	Pre-Intervention			Post-Intervention			Change				Pre-Intervention			Post-Intervention			Change				Baseline Group Comparison	Group × Time Effect	Overall Group Effect	Overall Time Effect
	Mean	±	SEM	Mean	±	SEM	Mean	±	SEM	*p*-Value *	Mean	±	SEM	Mean	±	SEM	Mean	±	SEM	*p*-Value *	*p*-Value	*p*-Value	*p*-Value	*p*-Value
Handgrip strength (kg)	29.6	±	3.2	34.2	±	3.6	4.6	±	1.1	<0.01	23.3	±	2.0	21.9	±	2.9	−1.4	±	2.2	0.54	0.14	0.03	0.03	0.21
Usual gait speed (m/s)	1.07	±	0.11	1.21	±	0.10	0.14	±	0.06	<0.05	0.92	±	0.09	0.91	±	0.08	−0.02	±	0.05	0.75	0.33	0.06	0.10	0.14
Chair Stands (number)	15.5	±	1.8	18.9	±	1.8	3.4	±	0.9	0.01	13.5	±	2.6	14.6	±	2.2	1.1	±	0.6	0.11	0.33	<0.01	0.31	<0.05
VO_2_max (ml/kg/min)	18.6	±	1.7	25.4	±	3.2	6.7	±	1.6	0.02	15.2	±	1.6	18.0	±	2.5	2.8	±	2.3	0.20	0.16	0.60	0.07	0.01

EX: exercise; BCAAs: branched-chain amino acids; PLA: placebo; SEM: standard error of the mean;

* Within-group pre- to post-intervention comparison.

## Data Availability

The data supporting the findings of this study are available from the corresponding author upon reasonable request. Due to privacy and ethical considerations, data cannot be shared publicly.

## Abbreviations

The following abbreviations are used in this manuscript:

BCAAs: Branched-chain Amino Acids
EX + PLA: Exercise combined with placebo group
EX + BCAAs: Exercise combined with BCAA group
BMI: Body Mass Index
MOCA: Montreal Cognitive Assessment
CES-D: Center for Epidemiological Studies Depression
DEXA: Dual-Energy X-ray Absorptiometry
ASMI: Appendicular Skeletal Muscle Index
CPET: Cardiopulmonary exercise test
HR: Heart Rate
FAS: Fatigue Assessment Scale
ISI: Insomnia Severity Index
VAS: Visual Analog Scale
mm: millimeters
RPE: Rate of Perceived Exertion
HR: Heart Rate Max
mTOR: Mammalian target of rapamycin
